# Programed death-1/programed death-ligand 1 expression in lymph nodes of HIV infected patients: results of a pilot safety study in rhesus macaques using anti–programed death-ligand 1 (Avelumab)

**DOI:** 10.1097/QAD.0000000000001217

**Published:** 2016-09-28

**Authors:** Amanda L. Gill, Samantha A. Green, Shahed Abdullah, Cecile Le Saout, Stefania Pittaluga, Hui Chen, Refika Turnier, Jeffrey Lifson, Steven Godin, Jing Qin, Michael C. Sneller, Jean-Marie Cuillerot, Helen Sabzevari, H. Clifford Lane, Marta Catalfamo

**Affiliations:** a CMRS/Laboratory of Immunoregulation, NIAID; b Laboratory of Pathology, NCI, NIH, Bethesda; c Clinical Support Laboratory, Leidos Biomedical Research, Inc.; d AIDS and Cancer Virus Program, Retroviral Pathogenesis Section, Leidos Biomedical Research, Frederick National Laboratory, Frederick; e Smithers Avanza Toxicology Services, Gaithersburg; f Biostatistics Research Branch, DCR, NIAID, NIH, Bethesda, Maryland; g EMD-Serono, Inc., Rockland; h Compass Therapeutics, Cambridge, Massachusetts, USA.; ∗ Amanda L. Gill and Samantha A. Green contributed equally to the article.

**Keywords:** anti–programed death-ligand 1 immunotherapy, HIV, HIV immunotherapy, programed death-1, programed death-ligand 1

## Abstract

The programed death-1 (PD1)/programed death-ligand 1 (PD-L1) pathway plays a critical role in balancing immunity and host immunopathology. During chronic HIV/SIV infection, there is persistent immune activation accompanied by accumulation of virus-specific cells with terminally differentiated phenotypes and expression of regulatory receptors such as PD1. These observations led us to hypothesize that the PD1/PD-L1 pathway contributes to the functional dysregulation and ineffective viral control, and its blockade may be a potential immunotherapeutic target.

**Methods:**

Lymph node biopsies from HIV-infected patients (*n* = 23) were studied for expression of PD1 and PD-L1. In addition, we assessed the safety and biological activity of a human anti-PD-L1 antibody (Avelumab) in chronically SIV-infected rhesus macaques.

**Results:**

PD-L1 expression was observed in cells with myloid/macrophage morphology in HIV-infected lymph nodes. Administration of anti-PD-L1 was well tolerated, and no changes in body weights, hematologic, or chemistry parameters were observed during the study. Blockade of PD-L1 led to a trend of transient viral control after discontinuation of treatment.

**Conclusion:**

Administration of anti-PD-L1 in chronic SIV-infected rhesus macaques was well tolerated. Overall, these data warrant further investigation to assess the efficacy of anti-PD-L1 treatment on viral control in chronic SIV infection as a prelude to such therapy in humans.

## Introduction

The programed death-1 (PD1)/programed death-ligand 1 (PD-L1) pathway plays a critical role in regulating immune responses against pathogens by controlling the balance between immunity and limiting tissue damage. In chronic viral infections such as HIV/SIV, hepatitis B virus, and hepatitis C virus, failure to completely eliminate virus leads to a sustained inflammatory/activated environment accompanied by the accumulation of T cells with diminished in-vitro effector function, ‘exhausted T cells’ [1–3]. In HIV/SIV infections, chronic T-cell immune activation is reflected by increased expression of a variety of immunomodulatory receptors including, PD1, CTLA-4, LAG3, CD244/2B4, CD160, and others [4–11]. In-vitro and in-vivo studies of HIV/SIV infection have shown that blockade of PD1 is associated with enhanced virus-specific responses and improved viral control [12–15]. These observations support the hypothesis that PD1/PD-L1 interactions contribute to the functional dysregulation, exhaustion, and ineffective viral control [8,16–18]. This evidence suggests that the PD1/PD-L1 pathway may be a potential target for immune intervention in patients with HIV infection.

PD1 is expressed upon activation by T, B, and natural killer (NK) cells, dendritic cells, and activated monocytes, although its function in the latter is not well defined [19]. PD1 interacts with two ligands, PD-L1 and PD-L2. PD-L1 is mainly expressed by hematopoietic cells, such as T and B cells, dendritic cells, macrophages, and some epithelial cells (lung and vascular endothelium) [19–22]. In contrast, PD-L2 expression is more restricted and is inducible in dendritic cells, macrophages, bone marrow–derived mast cells, and some B cells [19,23].

PD1 interaction with its ligands regulates T cell receptor signaling by distinct mechanisms leading to diminished effector function, including cytokine secretion, proliferation, cytotoxicity, motility, and cell survival [24–27]. In addition, engagement of PD1 with its ligands induces reverse signaling on PD-L1/PD-L2 expressing cells [19,28,29].

Lymphoid organs are the primary targets of HIV/SIV infection [30–33]. In HIV/SIV-infected lymph nodes, the germinal center (GC) T follicular helper CD4^+^ T cells (CD4^+^ Tfh) exhibit higher expression of PD1 compared with extra follicular CD4^+^ and CD8^+^ T cells. PD-L1 expression has been observed inside and outside germinal centers [34,35].

To obtain further insights as to the potential role of PD-L1 blockade in the treatment of HIV infection, we analyzed the expression of PD1 and PD-L1 in human lymph node from 23 patients infected with HIV. In addition, we have evaluated the safety and the effect on SIV viral load of in-vivo PD1/PD-L1 blockade using a fully human anti-PD-L1 mAb (MSB0010718C, Avelumab, EMD-Serono, Inc., Rockland, Massachusetts, USA) in rhesus (Rh) macaques chronically infected with SIV_mac239,_ that had previously received IL-15 [36].

## Materials and methods

### Patient samples

Patient characteristics are described in Supplementary material and methods and Table S1.

### Study design

Six chronically SIV-infected Rh macaques received weekly doses of saline (*n* = 3) or a mAb anti-PD-L1 (Avelumab) at a dose of 20 mg/kg (*n* = 3). At week 24, all treatments were discontinued, and animals were followed for 10 additional weeks (Supplementary material and methods, Fig. S1 and Table S2).

## Results

### Expression of programed death-1/programed death-ligand 1 in human lymph nodes from patients with HIV infection

**Fig. 1:**
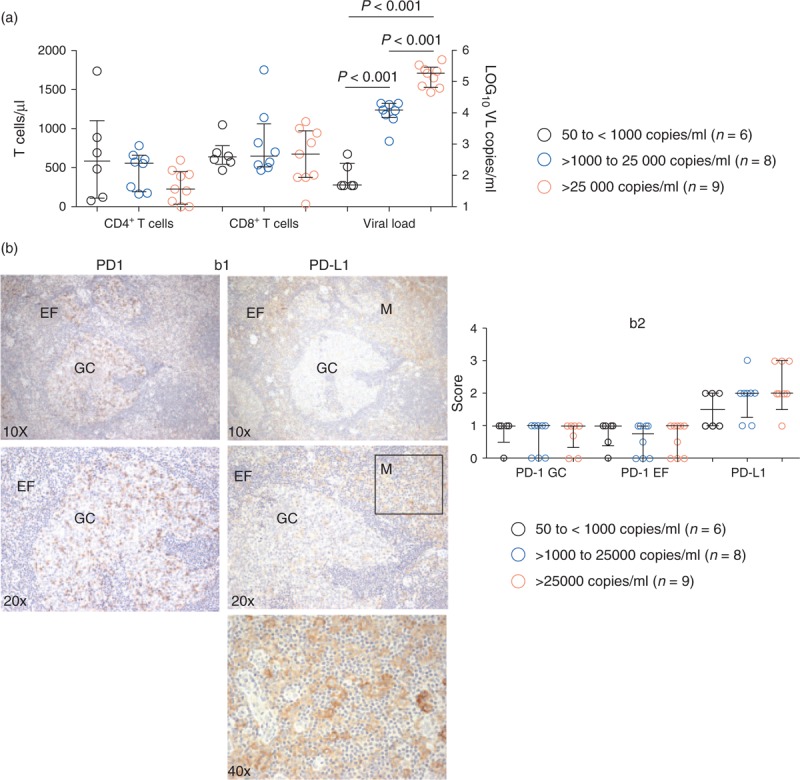
Expression of programed death-1 and programed death-ligand 1 in human lymph nodes from HIV-infected patients.

During HIV/SIV infection, there is an increase in PD1 expression on CD4^+^ and CD8^+^ T cells in peripheral blood [8,10]. To determine the distribution of PD1 and PD-L1 expression in lymphoid organs, we studied a cohort of HIV-infected patients (*n* = 23) that had previously undergone a lymph node biopsy. These patients had a median of 1 year [interquartile range: 0–4 years] of diagnosed infection (Supplementary Table S1 and Fig. 1a). For analysis, the patients were divided into three groups on the basis of viral loads, and the groups showed similar CD4^+^ and CD8^+^ T cell counts (Fig. 1a). Lymph nodes were stained with mAbs against PD1 and PD-L1 and blind-scored for expression of PD1 inside the germinal center and outside the germinal center (extra follicular). The score used for expression levels was negative (−), positive/negative (+/−), weakly positive (w+), and positive (+, ++, and +++). These scores correspond to 0, 0.5, 0.7, 1, 2, and 3, respectively in Fig. 1b. As reported by other groups [34,37], PD1^high^ cells were found within the germinal center and presumably represent CD4^+^ Tfh cells. PD1^+^ lymphoid were also found scattered in the extra follicular region (Fig. 1b, b1). PD-L1 expression was extra follicular and largely restricted to cells with myloid/macrophage morphology using this scoring system (magnified black square, Fig. 1b, b1). There were no significant correlations between expression of PD1 or PD-L1 and the levels of viremia (Fig. 1b, b2). Examination of longitudinal specimens from these patients, obtained 2, 6, and more than 24 months after the first biopsy, showed no changes in PD1 or PD-L1 expression despite viral suppression (data not shown). These results suggest that during HIV infection circulating PD1-expressing CD4^+^ and CD8^+^ T cells will likely encounter PD-L1 expressing cells in lymphoid organs.

(a) HIV-infected patients (*n* = 23) were divided on the basis of viral loads at the time of the lymph nodes biopsy. CD4^+^ and CD8^+^ T-cell counts are shown on the left *y*-axis. Viral load is shown on the right *y*-axis. Nonparametric Mann–Whitney test was used for the group comparisons, *P* value less than 0.05 was considered significant. (b) Lymph nodes biopsies (*n* = 23) were blind scored for programed death-1 expression inside the germinal center, outside the germinal center (GC), and in extra follicular (EF) regions, and programed death-ligand 1 expression was assessed outside the germinal centers in myloid/macrophage (M) morphology cells: shown in panel b1. The score used for expression levels was negative (−), positive/negative (+/−), weakly positive (w+), and positive (+, ++, and +++). These scores correspond to 0, 0.5, 0.7, 1, 2, and 3, respectively: shown in panel b2.

### Administration of a human anti–programed death-ligand 1 antibody to chronically SIV-infected rhesus macaques leads to a trend of transient virologic control

**Fig. 2:**
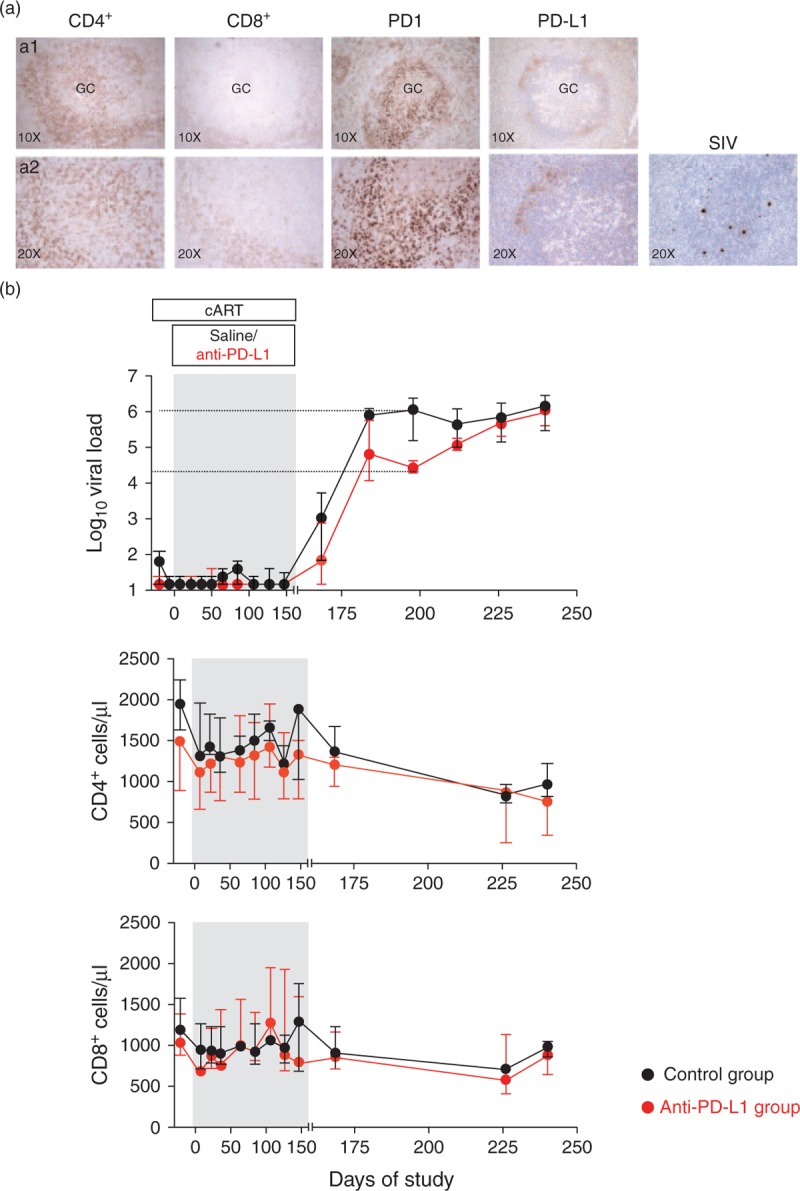
Delay in the viral rebound after cART discontinuation in animals treated with anti–programed death-ligand 1.

To test the hypothesis that blockade of the PD1/PD-L1 pathway may facilitate virologic control during HIV/SIV infection, we studied SIV-infected Rh macaques. We first examined spleens from Rh macaques infected with SIV_mac239_ for tissue distribution of PD1, PD-L1, and SIV-RNA. As previously described [34,37] and similar to observations in human lymph nodes (Fig. 1a), PD1^high^ cells were localized within the germinal center overlaying with CD4^+^ staining. Outside the germinal center (extra follicular), PD1 expression was dimmer and scattered. In contrast, PD-L1 expression was scattered within the germinal center and largely localized to cells with myeloid/macrophage morphology outside the germinal center (Fig. 2a).

(a) Spleens from chronically SIV_mac239_-infected rhesus macaques were stained for CD4^+^, CD8^+^, programed death-1, and programed death-ligand 1 expression. Magnification images (a1: 10×; a2: 20×). SIV_mac239_ was detected by RNAscope (a2). (b) Measurements of CD4^+^ and CD8^+^ T-cell counts and viral load during study. The control group (*n* = 3) is represented by black symbols, and the anti–programed death-ligand 1 treatment group (*n* = 3) is represented by red symbols. The *x*-axis represents days of the study, the left side: Days –30 to 161, and right side: Days 161–250. Day 161 indicates interruption of anti–programed death-ligand 1 and cART.

We next investigated the effects of in-vivo blockade of PD1/PD-L1 using a fully human anti-PD-L1 mAb in SIV_mac239_-infected Rh macaques with combination antiretroviral therapy (cART)-suppressed viremia. The animals received 24 weekly doses by intravenous infusion of saline for the control group (*n* = 3) or 20 mg/kg of anti-PD-L1 (*n* = 3) for the treatment group (Supplementary Fig. S1). After administration of the 24th dose (Day 161), both cART and anti-PD-L1 were discontinued, and viral loads, CD4^+^, and CD8^+^ T cell counts were monitored for an additional 10 weeks (Fig. 2b and Supplementary Table S2). At Day 169, animals had viral loads of 1.8, 2.9, and 1.2 copies/ml in the treatment group compared with 3.7, 3.0, and 1.8 copies/ml in the control group. Following an additional 2 weeks, SIV levels were 4.4, 4.4, and 4.6 copies/ml in the treatment group compared with 6.0, 6.4, and 5.2 copies/ml in the control group (Supplementary Table S2). The comparison of the area under the curve between the groups after treatment discontinuation (Days 161–240) did not reach significance (Fig. 2b, *P* = 0.14). We found no significant changes in CD4^+^ or CD8^+^ T cell counts between the treatment groups (Fig. 2b). In addition, no changes were observed in the expression of PD1 or PD-L1 by T, B, NK cells, and monocytes nor in T-cell cytokine secretion (Supplementary Figs. S2–S5). Expression of PD-L1 in splenic cells was higher than that observed in cells from peripheral blood mononuclear cells (Fig. S5). These results support the design of larger studies with greater power to assess the efficacy of anti-PD-L1 on SIV viral control to determine its potential use in HIV-infected patients.

## Discussion

In the present study, we describe the expression and spatial distribution of PD1 and PD-L1 in human lymph nodes from HIV-infected patients. In addition, we have assessed the safety and effect of a human anti-PD-L1 mAb on viral load in chronically SIV-infected Rh macaques. Administration of anti-PD-L1 was well tolerated, and no changes in body weights, hematologic, or chemistry parameters were observed during the study. We found that administration of anti-PD-L1 led to a trend of transient viral control after discontinuation of antiretroviral therapy. These data support larger studies to better assess the efficacy of anti-PD-L1 administration on viral control to evaluate its potential use in HIV-infected patients.

In chronic viral infections such as HIV/SIV, antigen persistence drives immune activation leading to an accumulation of terminally differentiated virus-specific cells in association with failure to achieve virologic control. Among the main characteristics of these virus-specific cells are increased expression of regulatory markers (PD1, CTLA-4, Tim3, and CD160) and diminished in-vitro effector function in terms of polyfunctional cytokine secretion, cytotoxicity, and proliferative capacity [8,10,16,17,38–40]. The PD1/PD-L1 pathway plays a central role in host defense, balancing pathogen elimination and limiting host immunopathology. Even in the setting of suppressed viral replication by cART, the immune systems of HIV-infected patients are unable to eliminate residual virus. This is associated with ongoing chronic immune activation as reflected by increased expression of PD-1 and PD-L1 [17,41,42]. It is postulated that modulation of this pathway may lead to better virologic control by promoting CD8^+^ T-cell expansion.

In the lymphoid organs, the primary targets of HIV infection, PD1 and PD-L1 are differentially expressed with specific spatial distribution and cell expression. PD1^high^ cells, corresponding mainly to CD4^+^ Tfh cells, are localized within the germinal center where they presumably interact with PD-L1 expressing B cells [34,37]. In contrast, circulating PD1^+^ T cells will interact with extra follicular PD-L1 expressing cells, such as myloid cells. In addition, our finding of a trend in virologic control associated with administration of anti-PD-L1 is consistent with previous observations in which administration of anti-PD1 to chronically SIV-infected nonhuman primates enhanced proliferation and function of virus-specific CD8^+^ T cells [12]. The enhanced anti-SIV response by anti-PD1 administration led to better virologic control in animals at an early phase of infection despite high levels of viremia. This effect was transient in those animals at later stages of chronic infection [12]. In addition, anti-PD1 treatment was reported to enhance memory B cell survival and humoral responses against SIV and to reduce immune activation [12,13,15]. Similar to the present study, administration of anti-PD-L1 (BMS-936559) to Rh macaque chronically infected with SIV_mac239_ led to a delay in viral rebound after discontinuation of cART, whereas the precise mechanisms are unclear [43]. In the present study, two of the animals in each group previously received infusions of IL-15 (Table S2), and the potential impact of this prior treatment on the present results will require further investigation. A recent study of a single-dose administration of anti-PD-L1 mAb (BMS-936559, 0.3 mg/kg) in 6 HIV-infected participants on cART and HIV-1 RNA of less than 40 and at least 0.4 copies/ml by single-copy assay (SCA) showed that treatment was well tolerated, and there was a trend toward an increased proportion of Gag-specific CD8^+^ T cells in two individuals. No changes were noted in the SCA over the 28 days postinfusion. This study was discontinued because of retinal toxicity in the animal studies [44].

The PD1/PD-L1 pathway plays a critical role in balancing immunity against pathogens and host immunopathology. During HIV/SIV infection, T cells and virus-specific cells express PD1 even in the context of suppressed viremia. The present data support further consideration of the blockade of this pathway as immunotherapy for chronic HIV infection.

## Acknowledgements

We thank the patients of the National Institute of Allergy and Infectious Diseases HIV Clinic for their participation. The SIV_mac239_ Gag Peptide Pool was obtained through the NIH-AIDS Reagent Program, Division of AIDS, National Institute of Allergy and Infectious Diseases, NIH. Some mAbs were provided by the NIH-Nonhuman Primate Reagent Resource. This work was supported by the Intramural Research Program of the National Institute of Allergy and Infectious Diseases, NIH. Leidos Biomedical Research, Inc. has been funded in whole or in part with federal funds from the National Cancer Institute, NIH, under contract no. HHSN261200800001E. The content of this publication does not necessarily reflect the views or policies of the Department of Health and Human Services, nor does mention of trade names, commercial products, or organizations imply endorsement by the U.S. Government.

### Conflicts of interest

There are no conflicts of interest.

## Supplementary Material

Supplemental Digital Content
