# Supplementary material for: Programed death-1/programed death-ligand 1 expression in lymph nodes of HIV infected patients: results of a pilot safety study in rhesus macaques using anti–programed death-ligand 1 (Avelumab)
Source: AIDS. 2016 Sep 28;30(16):2487–93. doi: 10.1097/QAD.0000000000001217 (PMC5051527; doi:10.1097/QAD.0000000000001217)
Supplement: Supplemental Digital Content [file aids-30-2487-s001.docx]

**Supplementary Data**

**Supplementary Figure S1.** Study design.

**Figure S2. T cell counts and immunophenotype during anti-PD-L1 treatment. (A)** Flow cytometry gating strategy of CD4 and CD8 T cell subsets in PBMCs (upper panel). CD4 and CD8 T cell counts during the study (lower panel). **(B)** Proportion of PD1^+^ or Granzyme B^+^ CD4 or CD8 T cell subsets in PBMCs during the study. Representative contour plots of PD1 and granzyme B expression (gray) and overlaid in blue full minus one (FMO). In **(A)** and **(B)** the control group (n=3) is represented by black symbols and the anti-PD-L1 treatment group (n=3) is represented by red symbols.

**Figure S3. Cytokine secretion after cART discontinuation in anti-PD-L1 treated animals. (A)** Gating strategy of cytokine secretion by CD4 and CD8 T cells. **(B)** Proportion of IL-2^+^IFNγ^+^, IL-2^+^TNFα^+^ and TNFα^+^IFNγ^+^ CD4 and CD8 T cells stimulated with a pool of SIV_mac239_ Gag-peptides (2μg/ml). **(C)** Proportion of IL-2^+^IFNγ^+^, IL-2^+^TNFα^+^and TNFα^+^IFNγ^+^ CD4 and CD8 T cells stimulated with SEB (2.5 μg/ml).

**Figure S4. Expression of PD1 and PD-L1 in PBMCs during anti-PD-L1 treatment.**

Flow cytometry analysis for PD1 and PD-L1 expression during study weeks -1 (pre-treatment), 12, 24, and 34. **(A)** Gating strategy for T, B and NK cells (upper panel). Proportion of PD1^+^ or PD-L1^+^ T, B, and NK cells **(B)** Gating strategy and proportion of PD-L1^+^ monocytes. Quadrants (A) and gates (B) were set up based on FMO controls. In both **(A)** and **(B)** the control group (n=3) is represented by black symbols and the anti-PD-L1 treatment group (n=3) is represented by red symbols.

**Figure S5. Expression of PD1 and PD-L1 in spleens after discontinuation of treatment (anti-PD-L1 and cART).** Spleens were harvested from RM a month after the end of the study and cells were analyzed by flow cytometry. **(A)** Flow cytometry gating strategy (T, B and NK cells) and analysis of the proportion of PD1^+^ or PD-L1^+^ in Total, CD4 T cells, and CD8 T cells. **(B)** Analysis of the proportion of PD1^+^ or PD-L1^+^ NK cells. **(C)** Analysis of the proportion of PD1^+^ or PD-L1^+^ B cells. **(D)** Flow cytometry gating strategy and analysis of the proportion PD-L1^+^ monocytes (Lin^+^CD14^+^) and myeloid cells (Lin^-^CD14^-^). Quadrant and gates were set up based on FMO staining controls. **(A-D)** Control group (n=3) is represented by black symbols and the anti-PD-L1 treatment group (n=3) is represented by red symbols.

**Supplementary Materials and Methods**

*Patient Samples:* The human study was conducted according to the principles expressed in the Declaration of Helsinki. LN biopsies from HIV infected patients were obtained under a NIAID Institutional Review Board-approved HIV clinical research study protocol in the NIAID/CCMD intramural program. Patients provided written informed consent for the collection of samples. Patient characteristics and sample analysis are described in Supplementary Material and Methods and Table S1*.*

*Immunohistochemical (IHC) staining on formalin-fixed, paraffin-embedded (FFPE) tissue:* Immunohistochemical staining on formalin-fixed, paraffin-embedded tissue sections was performed using a PD-L1 rabbit mAb (Cell Signaling, clone E1L3N, 1:200 dilution). After deparaffinization, antigen retrieval was performed (Target retrieval solution, high pH, Dako) and the slides were incubated overnight at room temperature. The detection was performed according to the manufacturer’s instructions using Signal Stain Boost IHC Detection Reagent HRP Rabbit (Cell Signaling) with DAB as a chromogen. A different rabbit monoclonal PD-L1 antibody was used for RM tissues (Abcam, clone EPR1161). In addition, on selected cases we used the following antibodies: mouse monoclonal anti-PD-1 (Abcam, clone NAT105), rabbit monoclonal anti-CD4 (Abcam, clone EPR6855), and anti-CD8 (Thermo Scientific, clone SP16). The detection was performed with UltraView Universal DAB Detection Kit (Ventana Medical Systems, Tucson, AZ) with an automated system (BenchMark XT, Ventana, Tucson, AZ).

*Animals and Study design:* Six female RM 18.5 months following SIV infection were studied under an approved protocol in accordance with US NIH guidelines, the USDA Animal Welfare Act, the PHS Policy on Humane Care and Use of Laboratory Animals, and the U.S. Interagency Research Animal Committee Principles for the Utilization and Care of Research Animals. All RM were previously infected intravenously with SIV_mac239_ (generously provided by Dr. JD Lifson, NCI, Frederick) and under a cART regimen (Emtricitabine at 30 mg/kg and Tenofovir at 20 mg/kg via daily subcutaneous injection, and Raltegravir at 400 mg/kg p.o twice/day). Prior treatments and viral load history are detailed in Supplementary Table S2. The Six animals (all of which were on cART and suppressed viremia for 16.5 months and four of which had previously received rhIL-15) were randomly assigned to two groups each consisting of three monkeys (each group included two animals with prior rhIL-15 treatment and one animal without prior rhIL-15 treatment). The animals were administered sterile saline (n=3) or a fully human anti-PD-L1 (MSB0010718C, Avelumab, EMD-Serono) at a dose of 20 mg/kg per dose per week (n=3). Administration of saline or anti-PD-L1 treatments was performed by continuous infusion via the saphenous vein for 20 ｱ 5 minutes. All animals continued to receive cART throughout the 24-week treatment period. At 24 weeks, all treatment and drug regimens were discontinued and animals were followed for 10 additional weeks (Supplementary Figure S1). Blood was collected from all animals for SIV viral load assessment (Hansen SG. et al. Nature. 2013), hematology, clinical pathology, and CD4 T cell counts.

*SIV detection in spleen cells by RNAscope:* RNAscope HybEZ Oven was used to detect SIV mRNA on FFPE RM spleens. The procedure was done using a SIV_mac239_ probe (Advanced Cell Diagnostics, P/N 312811) according to the manufacturer’s protocol. A negative probe (DapB, P/N 310043) and a positive probe (PPIB, P/N 313901) were used as controls.

*Flow Cytometry:* Heparinized whole blood was stained at room temperature for 15 minutes with the following mAbs to detect T cell subsets: anti-CD45 V450 (BD, D058-1283), anti-CD3 APC-Cy7 (BD, clone SP34-2), anti-CD4 PE-Cy7 (BD, clone SK3), anti-CD8 APC (BD, RPA-T8), anti-CD28 PerCP-Cy5.5 (BD, clone CD28.2), and anti-CD95 FITC (BD, clone DX2), anti-PD1 PE (BD, Clone EH12.1) and anti-Granzyme B (Life Technologies, Clone GB11). After staining, blood was incubated at room temperature for 10 minutes with FACS Lysing Solution (BD), and washed with PBS before acquisition. Samples were acquired on a BD LSR II and analyzed with FlowJo.

Flow cytometry analysis of frozen PBMCs and cells from spleens; cells were thawed and rested overnight in media. Cells were stained with Live/Dead fixable blue dead cell stain (Invitrogen) for 30 minutes on ice. After two additional washes, cells were incubated on ice for 10 minutes with 10 μg human IgG (Sigma-Aldrich) to block potential Fc receptor binding and then stained for 30 minutes with the following mAbs: for T, B, NK cells from PBMCs, anti-CD3 BV655 (BD, Clone SP34.2), anti-CD20 APC (BD, Clone 2H7), anti-PD1 APC-Cy7 (Biolegend, Clone EH12.2H7), anti-PD1 APC-Cy7 (Biolegend, Clone EH12.2H7), and anti-PD-L1 PE-Cy7 (BD, Clone MIH1). For monocytes from PBMCs, anti-CD2 PerCP-Cy5.5 (Biolegend, Clone RPA-2.10), anti-CD3 BV655 (BD, Clone SP34.2), anti-CD20 Pacific Blue (Biolegend, Clone 2H7), anti-CD14 APC (Biolegend, Clone M5E2) and anti-PD-L1 PE-Cy7 (BD, Clone MIH1) were used. Staining of T, B, and NK cell compartments from spleen utilized anti-CD3 BV655 (BD, Clone SP34.2), anti-CD4 PerCP (BD Pharmigen, Clone L200), anti-CD20 APC (BD, Clone 2H7), anti-PD1 APC-Cy7 (Biolegend, Clone EH12.2H7), and anti-PD-L1 PE-Cy7 (BD, Clone MIH1). For monocyte/myeloid compartment the following panel was used, anti-CD2 PerCP-Cy5.5 (Biolegend, Clone RPA-2.10), anti-CD3 BV655 (BD, Clone SP34.2), anti-CD20 Pacific Blue (Biolegend, Clone 2H7), anti-CD14 APC (Biolegend, Clone M5E2), anti-Lin1 FITC (BD), and anti-PD-L1 PE-Cy7 (BD, Clone MIH1) Samples were acquired on a BD LSR II and analyzed with FlowJo.

*Cytokine Secretion Assay:* Frozen PBMCs from control and anti-PD-L1 groups were re-suspended at 2x10^6^ cells/ml in X-VIVO 15 medium (Lonza) and rested overnight at 37°C. Cells were incubated with anti-CD28/49d (1 μg/ml; BD Biosciences) and either SEB (2.5 μg/ml) or SIV_mac239_-Gag peptide pool (2 μg/ml; NIH-AIDS Reagent Resource) for 2 hours at 37°C. After 2 hours, BFA (20 μg/ml) was added to each well and cells were incubated for an additional 4 hours at 37°C. Cells were harvested and stained with Live/Dead fixable blue dead cell stain (Invitrogen) for 30 minutes on ice. After two additional washes, cells were incubated on ice for 10 minutes with 10 μg human IgG (Sigma-Aldrich) to block potential Fc receptor binding and then stained for 30 minutes with anti-CD3 PerCP (BD, clone SP34.2), anti-CD4 Qdot 605 (NHP Reagent Resource, clone 19THY-5D7), anti-CD8 eVolve 655 (eBioscience, clone RPA-T8), anti-IFN-γ Pacific Blue (Biolegend, clone 4S.B3), anti-IL-2 FITC (Biolegend, clone MQ1-17H12), and anti-TNFα APC (BD, clone MAB11). Samples were acquired on a BD LSR II and analyzed with FlowJo.

**Table S1. Patient characteristics**

| **Patient characteristics** | | | | | | | | | | | | **Lymph node biopsies** | | |
| --- | --- | --- | --- | --- | --- | --- | --- | --- | --- | --- | --- | --- | --- | --- |
| **Patients** | **HIV infection (years)** | **cART (months)** | **LOG10 VL** | **CD4**  **cells/μl** | **CD8**  **cell/μl** | **CD4DR**  **(%)** | **CD4CD25 (%)** | **CD4CD38 (%)** | **CD8DR**  **( %)** | **CD8CD25**  **(%)** | **CD8CD38**  **(%)** | **PD-1 GC^#^** | **PD-1^#^ Extra-**  **follicular** | **PDL-1^#^**  **Myloid/**  **Macro-**  **phages** |
| **All patients**  **n= 23** | 1.0  (0, 4) | 0  (0, 24) | 4.3  (2.7, 5.1) | 420  (162, 616) | 646.0  (474, 941) | 5  (3, 6.3) | 6.0  (2.8, 10) | 17.50  (8.3, 23.8) | 26.50  (15, 36) | 2  (1, 2) | 43.5  (23.3, 49.8) | 1  (0, 1) | 1  (0, 1) | 2  (1, 2) |
| **n= 6**  **VL**  **50- <1000**  **(copies/ml)** | 4  (1.5, 6) | 38.5  (18.5, 62.5) | 1.7  (1.7, 2.4) | 592  (111, 1100) | 628  (553, 782) | 5.5  (3, 6.3) | 10.5  (2, 20.8) | 23.5  (5.5, 28.5) | 15  (11.5, 25.8) | 2  (1.75, 2) | 23  (20.5, 46.3) | 1  (0.5-1) | 1  (0.375-1) | 1.5  (1-2) |
| **n= 8**  **VL**  **>1000-25000**  **(copies/ml)** | 1.5  (0, 4) | 0  (0-0) | 4.1  (3.9, 4.3) | 561  (193, 654) | 643  (511, 1064) | 9  (6, 10) | 7  (4, 7) | 21  (13, 23) | 28  (24, 36) | 2  (1, 2) | 44  (27, 49) | 1  (0-1) | 0.75  (0-1) | 2  (1.3-2) |
| **n= 9**  **VL**  **>25000**  **(copies/ml)** | 0  (0, 0.5) | 0  (0, 0) | 5.27  (4.8, 5.5) | 229  (36, 445) | 677  (378, 973) | 3  (1.5, 5) | 3  (0.5, 8) | 11  (3, 23) | 32  (16, 41) | 2  (1, 3.5) | 48  (28, 54) | 1  (0.35-1) | 1  (0-1) | 2  (1.5-3) |

^#^PD1 GC: PD1 Germinal Center, PD1 and PD-L1. Expression of PD1 and PD-L1 in lymph nodes was blind scored and the score used was: negative (-), positive/negative (+/-), weakly positive (w+), and positive (+, ++, +++). These scores correspond to 0, 0.5, 0.7, 1, 2, 3, respectively.

Values between parentheses correspond to IQR

**Table S2. RM viral load history and previous interventions**

| **Rhesus Macaque #** | **Viral**  **Set-point Log_10_**  **(a)** | **IL-15 Cycles**  **(10 day infusion)** | | | | **Anti-PD-L1**  **(b)** | **Viral Load**  **Log_10_ (copies/ml)**  **(c)** | | | | | | |
| --- | --- | --- | --- | --- | --- | --- | --- | --- | --- | --- | --- | --- | --- |
|  |  | **1^st^** | **2^nd^**  **33 days** | **3^rd^**  **32 days** | **4^th^**  **33 days** | **24 wks** | **Day**  **148** | **Day**  **169** | **Day**  **184** | **Day 198** | **Day 212** | **Day 226** | **Day 240** |
| **21667** | 6.3 | - | - | - | - | - | 1.2 | 3.7 | 5.9 | 6.0 | 5.6 | 5.8 | 6.1 |
| **21668*** | 7.3 | + | + | + | + | - | 1.5 | 3.0 | 6.1 | 6.4 | 6.1 | 6.2 | 6.4 |
| **21669*** | 7.2 | + | + | + | + | - | 1.2 | 1.8 | 5.9 | 5.2 | 5.0 | 5.1 | 5.5 |
| **21670** | 6.9 | - | - | - | - | + | 1.2 | 1.8 | 4.0 | 4.4 | 5.0 | 5.8 | 6.0 |
| **21671*** | 7.0 | + | + | + | + | + | 1.2 | 2.9 | 4.8 | 4.4 | 4.9 | 5.3 | 5.6 |
| **21672*** | 6.7 | + | + | + | + | + | 1.2 | 1.2 | 5.7 | 4.6 | 5.3 | 5.7 | 6.0 |

(a) Viral set points and prior interventions (rhIL-15) that the animals in the study received. Animals were initially infected and viral loads were monitored until their viral set point was reached (approximately 2 months). Animals started cART and maintained suppressed viremia to <30 copies/ml for 16.5 months before initiation of anti-PD-L1 treatment.

(b) Anti-PD-L1 treatment started 7 months later (weekly dosing regime).

(c) Viral loads before and after discontinuation of anti-PD-L1 and cART, Day 148 and Day 169, respectively. Discontinuation of treatment occurred on Day 161.
